# Cationic Single-Chained Surfactants with a Functional Group at the End of the Hydrophobic Tail DNA Compacting Efficiency

**DOI:** 10.3390/pharmaceutics13040589

**Published:** 2021-04-20

**Authors:** José Antonio Lebrón, Pilar López-Cornejo, Elena García-Dionisio, Pablo Huertas, Margarita García-Calderón, María Luisa Moyá, Francisco José Ostos, Manuel López-López

**Affiliations:** 1 Departamento de Química Física, Facultad de Química, Universidad de Sevilla, C/Profesor García González 1, 41012 Sevilla, Spain; lebronjunior@hotmail.com (J.A.L.); pcornejo@us.es (P.L.-C.); elena_garcia93@hotmail.com (E.G.-D.); 2 Departamento de Genética, Universidad de Sevilla y Centro Andaluz de Biología Molecular y Medicina Regenerativa (CABIMER), Universidad de Sevilla-CSIC-Universidad Pablo de Olavide, 41092 Sevilla, Spain; phuertas@us.es; 3 Departamento de Bioquímica Vegetal y Biología Molecular, Facultad de Química, Universidad de Sevilla, C/Profesor García González 1, 41012 Sevilla, Spain; marbioq@us.es; 4 Departamento de Ingeniería Química, Química Física y Ciencias de Materiales, Facultad de Ciencias Experimentales, Universidad de Huelva, Campus de El Carmen, Avenida de las Fuerzas Armadas s/n, 21071 Huelva, Spain

**Keywords:** cationic surfactants, DNA compacting efficiency, liposomes, lipoplexes, gene transfection

## Abstract

The interaction between calf-thymus DNA, ctDNA, and various single-chained surfactants with different functional groups at the end of hydrophobic tail was studied with the goal of investigating the influence of the functional group nature on surfactant DNA compacting efficiency. The surfactants investigated were dodecyltriethylammonium bromide (DTEABr), triethyl(1-phenoxydodecyl)ammonium bromide (12PhBr), triethyl(2-naphthoxydodecyl)ammonium bromide (12NBr) and 11-(isonicotinoyloxy)-*N*,*N*,*N*-triethyl-1-undecanaminium bromide (11PyBr). Results made evident that the surfactants’ tendencies to self-aggregation is the key factor determining their efficiency to compact the nucleic acid. Subsequently, DOPE/12NBr/pEGFP-C1 lipoplexes, with different cationic surfactant molar fractions (α) and mass ratios (L/D), were prepared and characterized. DOPE is a zwitterionic phospholipid 1,2-dioleoyl-sn-glycero-3-phosphoethanolamine, and the plasmid pEGFP-C1 carries a GFP coding sequence with the necessary regulatory elements for constitutive expression of the gene in human cells. 12NBr was chosen because it was the most efficient DNA compacting agent among the surfactants investigated. Finally, the cytotoxicity and transfection efficiency (TE) of DOPE/12NBr/pDNA lipoplexes, with different compositions, were investigated.

## 1. Introduction

Research on the interactions between surfactants and nucleic acids has drawn great attention in the last decades for its significance in biomedical and biotechnological applications, particularly for the possibility of using surfactant/DNA systems for gene delivery [1,2,3,4,5,6,7]. Since it is difficult for DNA to cross negatively charged cell membranes due to its high negative charge and bulky size, the surfactant gene vectors should compact DNA molecules into small particles, protecting them from the degradation of nucleases. Subsequently, they penetrate through the membrane into the cells, where the surfactant/DNA complexes decompact to release the genetic charge [2,8]. On these bases, the study of the conformational changes of DNA induced by the interaction with surfactants is of great significance in developing practical technologies for biomedical engineering. Many methods have been used in the investigation of DNA compaction by surfactants, both involving a large number of molecules [9,10,11,12,13] and at a singular molecule level [14,15,16,17,18].

The characteristics of the surfactants strongly determine the surfactant/DNA interactions. Anionic, nonionic and cationic surfactants have been investigated and, due to the charge complementarity, cationic surfactants have been shown to be by far the most efficient DNA compacting agents, inducing a morphological transition of the nucleic acid from an elongated form to a globular form [1,3,4,5,7,12,13,19,20,21,22,23,24]. An increment in the positive charge of the surfactant head group, or in the number of the positively charged head groups, tends, a priori, to increase the surfactant DNA compacting efficiency due to an increment in the electrostatic attractions between the negatively charged DNA and the surfactant [25,26]. The influence of the counter ion nature, the effect of substituting H atoms by F atoms in the hydrophobic chains, and the impact of the magnetic properties of the surfactants on the surfactant/DNA interactions, have also been investigated [27,28,29].

The importance of hydrophobic interactions has been demonstrated, in addition to electrostatic effects, in the DNA-condensing capacity of surfactants. An increase in hydrophobic chain length and the presence of additional side hydrophobic tails, results in an improved compacting efficiency [16,30,31]. More favorable hydrophobic interactions also explain that, for a given tail length, dimeric surfactants are more efficient DNA-condensing agents than single-chained surfactants [1,4,5,25,26,27,28,32,33,34,35,36]. Spacer length, which is a structural characteristic specific for oligomeric surfactants, also influences the surfactant/DNA interactions [37,38,39,40,41]. Recently, the effects of the degree of oligomerizarion of surfactants, as well as of the structure of the spacer group linking the individual surfactant fragments, have been examined [42]. The conclusion was that the DNA compacting efficiency of these surfactants is not simply connected to their degree of oligomerization, but is the result of a complex balance of the number and relative distances of the positively charged head groups and/or hydrophobic chains in the surfactant, which makes the interaction with the nucleic acid more effective, rendering appropriate surfactant/DNA complexes.

**Scheme 1:**
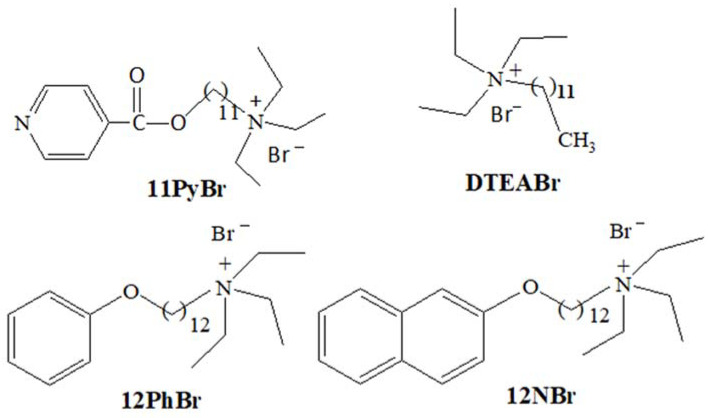
Structure of the surfactants investigated in this work.

Even though surfactant/DNA interactions have been studied in numerous papers, to the authors’ knowledge, the influence of the incorporation of a functional group at the end of the hydrophobic surfactant tail on the surfactant DNA compacting capacity has not been investigated. With this goal, the interactions between calf-thymus DNA, ctDNA, and the cationic surfactants 11-(isonicotinoyloxy)-*N*,*N*,*N*-triethyl-1-undecanaminium bromide (11PyBr), dodecyltriethylammonium bromide (DTEABr), triethyl(1-phenoxydodecyl)ammonium bromide (12PhBr), and triethyl(2-naphthoxydodecyl)ammonium bromide (12NBr) (see Scheme 1) were studied. The hydrophobicity of DTEABr, 12PhBr, and 12NBr increases following the trend DTEABr < 12PhBr < 12NBr because of the introduction of the phenoxy and naphthoxy groups at the end of the hydrophobic tail (the three surfactants have a 12-carbon chain). As a consequence, the expected trend in the cmc is cmc(DTEABr) > cmc(12PhBr) > cmc(12NBr). This would permit investigation of the influence of the surfactant tendency to self-aggregate on its DNA compacting capacity for surfactants with functional groups at the end of the hydrophobic tail. On the other hand, 11PyBr has an aromatic functional group at the end of the tail, but an 11-carbon chain. Therefore, information about the influence of the tail length on the surfactant/DNA interactions could be obtained. Subsequently, liposomes of different compositions containing DOPE (1,2-dioleoyl-sn-glycero-3-phosphoethanolamine) and the surfactant with the highest DNA compacting efficiency, 12NBr, were prepared. The formation and characterization of several DOPE/12NBr/pDNA lipoplexes, where pDNA is the plasmid pEGFP-C1, were done. Cytotoxicity of these lipoplexes in a human bone osteosarcoma epithelial U2OS cell line was studied, and then the transfection efficiency (TE) of these DOPE/12NBr/pDNA lipoplexes in the same cell line was investigated. These studies are relevant considering that, after a better understanding of the DNA compaction process by the surfactants studied, the search for more effective cationic surfactant nonviral vectors for gene therapy would be a priority research goal, given the number of rare disorders in the population which need medical solutions.

## 2. Materials and Methods

### 2.1. Materials

Red Safe was purchased from iNtRON (Biotechnologiy, Chicago, IL, USA). The lipid 1,2-dioleoyl-sn-glycero-3-phosphoethanolamine (DOPE) was obtained from Avanti Polar Lipids (Alabaster, AL, USA). The rest of the reagents were from Sigma Aldrich (Darmstadt, Germany) of the highest purity available, and used as received.

The pEGFP-C1 plasmid (Clontech, Biocientífica S.A., Buenos Aires, Argentina), pDNA, was extracted from competent *E. coli* bacteria previously transformed with pEGFP-C1. The extraction was done using a GenElute HP Select Plasmid Gigaprep kit (Sigma Aldrich, Darmstadt, Germany) following a protocol previously described [43]. FuGENE 6 was from Promega Corporation (Madison, WI, USA). ctDNA concentration was estimated using absorbance measurements at 260 nm (molar absorptivity 6600 mol^−1^ dm^3^ cm^−1^ [44]). An agarose gel electrophoresis test using ethidium bromide (EB) indicated that the average number of base pairs was above 10,000 bp [45]. The ctDNA concentration was expressed per base-pairs.

The syntheses of the cationic surfactants were previously described [46,47,48]. Their purity (≥99%) was checked by ^1^H and ^13^C NMR, elemental analysis and mass spectra.

MilliQ water (resistivity > 18 MΩ·cm) was used to prepare all solutions. The pH was maintained constant at 7.4 by using 10 mM HEPES (4-(2-hydroxyethyl)piperazine-1-ethanesulfonic acid sodium salt) buffer. All measurements were done at 303.0 ± 0.1 K.

### 2.2. Preparation of Surfactant/ctDNA Solutions

Surfactant/ctDNA solutions were prepared by using the following method. A stock ctDNA solution (~5 × 10^−4^ M) was prepared in buffer HEPES 10 mM, at pH = 7.4 and several aliquots were stored at −70 °C. The required amounts of the different surfactants were weighed in order to prepare stock surfactant solutions in HEPES 10 mM (pH = 7.4). Cationic surfactant/ctDNA solutions, with 3.0 × 10^−5^ M of ctDNA, at the desired charge ratio cationic surfactant/ctDNA (N/P) were prepared adding the required volumes of the stock solutions and using the buffer as solvent. All solutions were freshly prepared before use. In the case of the AFM experiments, solutions were prepared using the same method, but the ctDNA concentration was different (see Section 2.9).

### 2.3. Preparation of Lipoplexes

The lipid thin-film hydration method was used to prepare the liposomes [49]. Concisely, adequate quantities of surfactants and DOPE were dissolved in chloroform. Different volumes of these solutions were mixed in order to obtain the desired cationic lipid molar fraction, α, given by Equation (1):

(1) $$\alpha =\frac{n_{\mathrm{surfactant}}}{n_{\mathrm{surfactant}}+n_{\mathrm{DOPE}}}$$

where n_surfactant_ and n_DOPE_ are the mole number of the cationic surfactant and the zwitterionic DOPE, respectively, in the total volume of the organic solution.

The organic solvent was evaporated by using a rotary evaporator at 303 K for 50 min, obtaining a dry lipid film which was stored for at least 24 h at 193 K to avoid degradation [50]. Subsequently, the lipid film was hydrated with 2 mL of an aqueous buffered solution (HEPES 10 mM, pH = 7.4) and submitted to 10 cycles of vortex (3 min/1200 rpm) and sonic action (2 min, JP Selecta Ultrasons system 200 W, 50 kHz, Abrera, Barcelona, Spain). Finally, the solution was vortexed at room temperature for 2 h. The resulting liposomes were multilamellar, with a high polydispersity. In order to obtain unilamellar liposomes with a homogeneous size distribution, the liposome solutions were extruded 10 times with a mini extruder from Avanti Polar Lipids (Birmingham, AL, USA), using polycarbonate membranes of 100 and 200 nm (Whatman, Maidstone, UK). After extrusion, the solutions were kept in the dark at 277 K for 24 h to obtain complete stabilization.

The lipoplexes were prepared by mixing appropriate volumes of both liposome and aqueous HEPES 10 mM pDNA solutions in order to obtain the desired L/D values for each α value investigated. No dilution was used. The mass ratio L/D is defined by Equation (2):

(2) $$\frac{L}{D}=\frac{m_{\mathrm{surfactant}}+m_{\mathrm{DOPE}}}{m_{\mathrm{surfactant}}}$$

here m_DOPE_, m_surfactant_, and m_DNA_ are the masses of the zwitterionic phospholipid of the surfactant, and of the DNA, respectively, in the solution. In all the liposome solutions investigated, the mass of DNA was kept constant at 10^−4^ g (8.1 × 10^−5^ M given in base-pairs).

The stability of the DOPE/12NBr/pDNA lipoplexes was followed by changes in their size with time. The size remained unchanged for more than 48 h. The authors also checked the stability of DOPE/12NBr/pDNA lipoplexes of different compositions after dilution with buffer HEPES 10 mM. No variations in their size were observed.

### 2.4. UV-Visible Spectroscopy

UV-visible spectroscopy was used to check the stability of the surfactant/ctDNA solutions. A spectrophotometer Hitachi UV-visible 3900 (Chiyoda, Tokyo, Japan) was used. It was connected to a water flow Lauda cryostat (Stuttgart, Baden-Würtenberg, Germany) for controlling temperature, which was kept at 303.0 ± 0.1 K. No changes in absorbance were observed at 260 nm for more than 48 h in all systems investigated. Nonetheless, the surfactant/ctDNA solutions were freshly prepared before used.

### 2.5. Fluorescence Measurements

A Hitachi F-2500 spectrofluorimeter (Chiyoda, Tokyo, Japan) interfaced to a PC for the recording and handling of the spectra, and connected to a flow Lauda thermostat (Stuttgart, Baden-Würtenberg, Germany) to maintain the temperature at 303.0 ± 0.1 K, was used with a standard fluorescence quartz cell of 10 mm path length.

Ethidium bromide (EB) emission fluorescence intensity was measured using excitation and emission wavelengths of 520 and 588 nm, respectively. EB emission spectra were recorded from 530 to 700 nm with a scan rate of 60 nm/min and excitation and emission slits equal to 5 nm for both. The EB and ctDNA concentrations were kept constant at 4.9 × 10^−6^ M and 3.0 × 10^−5^ M, respectively, while changing the surfactant concentration. All the solutions studied were buffered (HEPES 10 mM, pH = 7.4). Data are expressed as mean±SD from at least three separate experiments, *n* = 3.

### 2.6. Zeta Potential Measurements

Zeta-potential, ζ, values were calculated measuring the electrophoretic mobility of both the surfactant/DNA and the DOPE/12NBr/pDNA solutions from the velocity of the particles using a laser Doppler velocimeter (LDV). The experiments were done with a Zetasizer Nano ZS Malvern Instrument Ltd. (Malvern, Worcestershire, UK) at 303.0 ± 0.1 K. DTS1060 polycarbonate capillary cells were used. DNA concentrations used were 3.0 × 10^−5^ M in the surfactant/DNA buffered solutions and 8.1 × 10^−5^ M in the buffered solutions of liposomes. Data are expressed as mean ± SD from at least three separate experiments, *n* = 9.

### 2.7. Dynamic Light Scattering (DLS) Measurements

A Zetasizer Nano ZS Malvern Instrument Ltd. (Worcestershire, UK) was used to estimate the size and the polydispersity index (PDI) of the DOPE/12NBr/pDNA lipoplexes using DLS measurements. A scattering angle of 90° with a helium-neon laser was utilized, and the apparatus analyzed the fluctuations in the intensities of the scattered light. A fixed concentration of 8.1 × 10^−5^ M of the plasmid pDNA was present in all the solutions investigated. Composition of the lipoplex solutions was changed, keeping constant different α values and varying the L/D ratio. Data are expressed as mean ± SD from at least three separate experiments, *n* = 10. Temperature was maintained at 303.0 ± 0.1 K.

### 2.8. Agarose Gel Electrophoresis

Agarose gel (1%) was prepared in a TAE buffer (1 mM EDTA, 40 mM Tris-acetate) in a total volume of 180 µL and stained with the dye Red-Safe (10 μL) for the visualization of the nucleic acid bands. The ctDNA concentration was kept constant at 3 × 10^−5^ M for the surfactant/ctDNA solutions while changing the surfactant concentration. In the case of the DOPE/12NBr/pDNA lipoplex solutions, the pDNA concentration was 8.1 × 10^−5^ M. Composition of the lipoplex solutions was changed by keeping constant the different α values and varying the L/D ratio. In each experiment, 20 μL of the DOPE/12NBr liposome solution were mixed with 5 μL of 5× DNA loading buffer. Then, after homogenization, the resulting solutions were added in each well. Electrophoresis was carried out for 90 min at 90 V. A detector Ultima 16si (Hoefer, Germany) was used for visualizing the nucleic acid bands by irradiation with UV light (254 nm).

### 2.9. Circular Dichroism Spectra

The circular dichroism (CD) spectra were recorded in a Biologic Mos-450 spectropolarimeter (Cambridge, UK). A standard quartz cell of 10 mm path length was utilized, and scans were taken from 220 to 310 nm. Three independent experiments were carried out and each spectrum was obtained from an average of 10 runs at a fixed temperature of 303.0 ± 0.1 K, with a 5 min equilibration before each scan. The ctDNA concentration was kept constant at 3.0 × 10^−5^ M, for the surfactant/ctDNA solutions while changing the surfactant concentration. The pDNA concentration remained constant at 8.1 × 10^−5^ M in the lipoplex solutions. For a given α value, the ratio L/D was changed. All solutions were prepared in 10 mM HEPES buffer at pH 7.4.

### 2.10. Atomic Force Microscopy, AFM

The structures of the surfactant/ctDNA complexes were investigated using AFM. A Molecular Imaging PicoPlus 2500 AFM (Agilent Technologies, Santa Clara, CA, USA) was used. A resonance frequency of around 240 KHz and a nominal force constant of 42 N/m were the working conditions, using silicon cantilevers (Model Pointprobe, Nanoworld, Neufchâtel, Switzerland). The images were recorded in air and in tapping mode. Data collection was registered at 256 × 256 pixels, with scan speeds about 0.5 Hz. The ctDNA concentration was 0.6 μM, keeping pH = 7.4 (HEPES 10 mM).

Images of surfactant/ctDNA buffered solutions were obtained using the following method. (i) 0.1% (*v*/*v*) APTES aqueous solution was dropped onto a freshly cleaved mica surface in order to prepare a modified mica surface. After 20 min, it was washed with ultra-pure water and air dried. (ii) A 30 μL droplet of the surfactant/ctDNA buffered solution was deposited on the modified mica surface and incubated for 30 min. (iii) Afterwards, it was washed with pure water and air dried for AFM imaging.

### 2.11. Electron Transmission Microscopy (TEM)

A Zeiss Libra 120 scanning electron microscope at 80 kV was used to characterize the DOPE/12NBr liposomes and the DOPE/12NBr/pDNA lipoplexes. A 300-mesh copper grid coated with collodion was impregnated with the samples and, subsequently, it was stained with a solution of uranyl acetate (2.0%). Images were processed with a bottom mounted TEM CCD camera and recorded with a resolution of 2048 × 2048 pixels. The pDNA concentration in the lipoplexes formed was 2.5 × 10^−5^ g/mL.

About 100 particles were taken into account from different images obtained for independent experiments corresponding to each studied system. ImageJ bundled with 64-bit Java 1.8.0_172 was used to analyze TEM images.

### 2.12. In Vitro Cytotoxicity Assays

The cytotoxicity of the lipoplexes with α 0.2, 0.3, and 0.7, at different L/D values, was estimated in vitro by using the MTT assay [51]. Cell lines were plated out into 96 well plates at a density of 7000 cells per plate. The U2OS cell line was used in order to evaluate the cytotoxicity of these lipoplexes. The next day, different doses of lipoplex solutions were added to the wells, keeping the pDNA amount constant at m_pDNA_ = 2 µg, and the plate returned to the incubator for two days more. Later, they were pulsed with MTT (ROCHE, Basilea, Switzerland). Cell viability was measured by luminometry according to the manufacturer instructions in Varioskan Flash (Thermo Electron Corporation) and normalized with control (FuGENE^®^ HD, Promega, E2311). Each L/D value investigated was measured in triplicate. The cell line used is from ATTC^®^ HTB-96^TM^ (ATCC, Manassas, VA, USA).

### 2.13. Transfection Assays

The plasmid pEGFP-C1, pDNA. was used for these experiments given that it carries an enhanced GFP coding sequence with the required regulatory elements for constitutive expression of the gene in human cells. The cell line chosen was the U2OS from human osteosarcoma because it is an easy-to-transfect cell line. For this reason, it has been frequently used in human molecular and cellular biology studies. The pDNA nonviral vectors used were DOPE/12NBr liposomes of different compositions. The following method was used to carry out the transfection experiments: 3 μg of pDNA was added to a solution containing 180 μL of Opti-MEM (Gibco) and the amount of liposome buffered solution (HEPES 10 mM) necessary to obtain the L/D ratio for each α value investigated. The resulting mixture was incubated at room temperature for 20 min and, subsequently, added to a 50% confluent 6 cm plate with 3 mL of DMEM medium.

As negative control, the cells were transfected only with a mixture of transfection reagent and Opti-MEM (not plasmid DNA included). As a positive control we used FuGENE^®^ HD transfection reagent (Promega, E2311) according to the manufacturer’s protocol (i.e., 3 μg of plasmid DNA in 200 μL Opti-MEM plus 9 μL of FuGENE^®^ HD). Transfection efficiency was evaluated by flow cytometry with a FACSCalibur (BD) 24 h after transfection.

### 2.14. Statistical Analysis

Values are expressed as the mean ± standard errors of separate experiments. Statistical analysis was performed with Student’s t-test and One-way analysis of variance (ANOVA). When *p* < 0.05 (95% confidence) the differences were considered as significant.

## 3. Results and Discussion

### 3.1. DNA Compacting Efficiency of the Surfactants

The surfactant concentration present in the solutions was always lower than the cmc in order to avoid the aggregation of the amphiphilic molecules. Table S1 (Supplementary Material) summarizes the cmc of the surfactants investigated in this work at 303 K, taken from [46,47,48]. The tendency to self-association follows the trend 12NBr > 12PhBr >> DTEABr. The cmc corresponding to 11PyBr is not listed in this table because this surfactant does not aggregate in aqueous solutions. The stability of the surfactants/ctDNA solutions was checked by registering the UV-visible spectra with time. In all cases neither absorbance changes at 260 nm nor turbidity were observed for at least 48 h.

**Figure 1:**
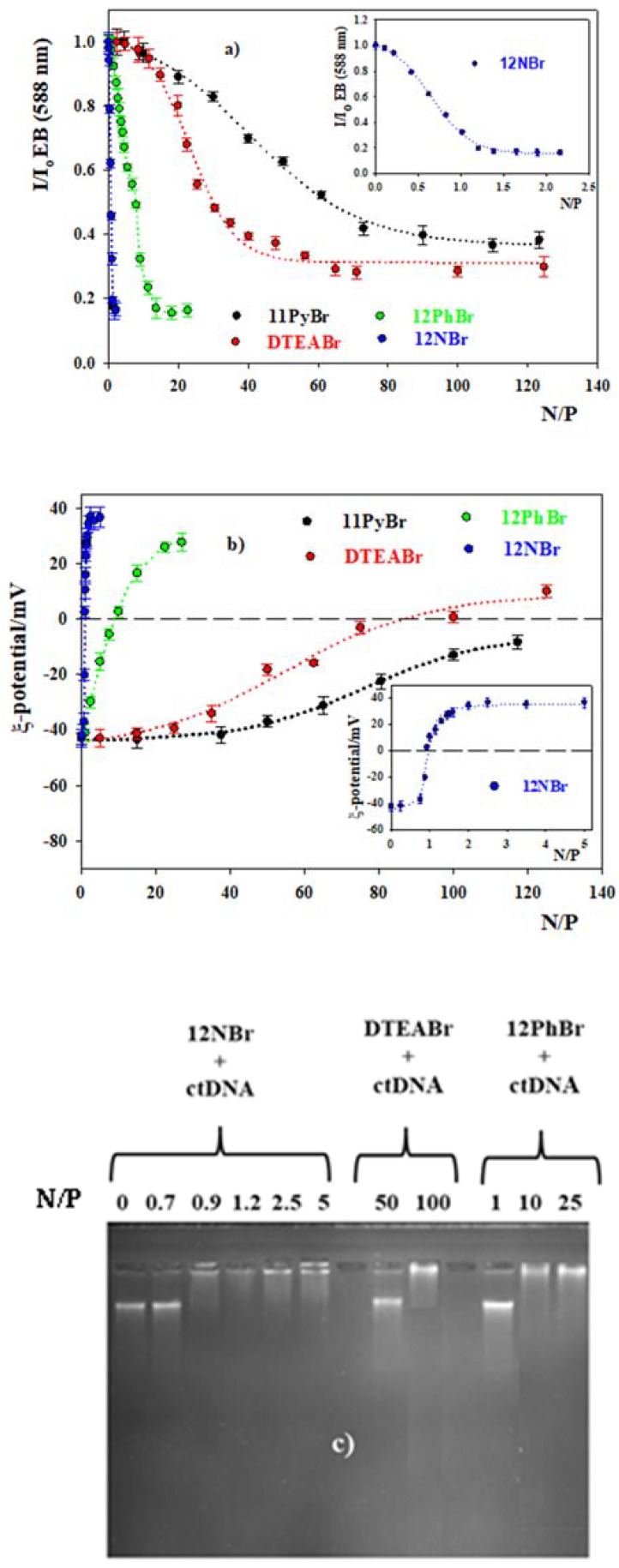
(**a**) Plots of I/Io as function of the charge ratio N/P. Experimental conditions: 10 mM HEPES (pH = 7.4), [EB]=2.4 × 10^−6^ M, [ctDNA] = 3.0 × 10^−5^ M, and T = 303.0 ± 0.1 K. (**b**) Plots of the variation of the zeta potential, ζ, with the charge ratio N/P, the experimental values are expressed as the mean ± SD (*n* = 9). Experimental conditions: [ctDNA] = 3.0 × 10^−5^ M, HEPES 10 mM (pH = 7.4) and T = 303.0 ± 0.1 K. (**c**) Electrophoretic mobility shift assay on an agarose gel (1%) for surfactant/ctDNA complexes. Experiments were run in TAE buffer (40 mM tris-acetate, 1 mM EDTA) and stained with Red Safe. Electrophoresis was performed at 90 V until completion. The insets in Figure 1a,b correspond to the 12NBr surfactant.

There are several methods for investigating the DNA compacting capacity of surfactants and other species. One of the easier and more frequently used is the ethidium bromide (EB) competitive assay [52,53,54]. EB is a planar dye that intercalates between adjacent base pairs of double stranded DNA, resulting in an enhancement of its fluorescence emission intensity. Changes in this magnitude when a surfactant is added to an EB/ctDNA solution can provide information about surfactant/ctDNA interactions. Figure 1a shows the dependence of I/Io on the N/P ratio. N/P = (1 × M_surfactant_)/(k × M_DNA_), where M_surfactant_ and M_DNA_ are molar concentrations of the surfactant and of the nucleic acid (expressed in base pairs), respectively, 1 is the charge of the surfactants investigated and k represents the quantity of negative charge attributable to the nucleic acid molecules per base pair, which is equal to 2 [55]. Io and I are the fluorescence emission intensities of EB in the absence and in the presence of surfactant, respectively. One can see in this figure that 12NBr and 12PhBr practically displaced all EB molecules from their intercalation site in ctDNA to the bulk solution. DTEABr and, particularly, 11PyBr only partially displaced the dye. Nonetheless, these results indicate that all the cationic surfactants studied interacted with the nucleic acid, the trend observed being 12NBr > 12PhBr >> DTEABr > 11PyBr. The displacement of the dye molecules to the bulk could be due to conformational changes caused by the surfactant/ctDNA interactions.

Conformational changes in nucleic acids are usually accompanied by a charge inversion. In order to investigate this point, zeta potential measurements, ζ were carried out in buffered surfactant/ctDNA solutions, keeping constant the nucleic acid concentration while varying the surfactant concentration. Figure 1b shows the dependence of ζ on the charge ratio N/P for the surfactants investigated. One can see in this Figure that 12NBr and 12PhBr caused a charge inversion of the nucleic acid, reaching a high positive charge. DTEABr/ctDNA complexes reached a low positive charge at high surfactant concentration, whereas 11PyBr was not able to provoke the charge inversion even at really high 11PyBr concentrations.These results point out the large differences in the interactions of the surfactants investigated with ctDNA. The charge inversion has previously been associated with the condensation and conformational changes of the nucleic acid in the surfactant/ctDNA complexes and it has been explained by an entropy increase, due to the release of the Na^+^ ctDNA counterions and the screened electrostatic interactions [38,56].

An additional way to investigate the charge inversion of nucleic acids caused by their interactions with other species is by using agarose gel electrophoresis. Figure 1c shows that for low enough surfactant concentrations, different for each surfactant, the bands of the surfactant/ctDNA solutions moved towards the anode, indicating that negatively charge nucleic acid was present. For 12PhBr and 12NBr when the charge ratio N/P increases, the migration of the ctDNA through the gel was completely hindered, making evident the charge inversion of the nucleic acid. For DTEABr the charge inversion was observed at high N/P values. No experiments were done for 11PyBr since this surfactant did not cause the charge inversion of ctDNA (see Figure 1b). These results are in agreement with those shown in Figure 1a,b.

Figure 1 indicates that there are strong interactions between ctDNA and 12PhBr and 12NBr. This interaction was weak with DTEABr and, particularly, with 11PyBr. Except for the latter, an inversion of the nucleic acid charge was observed, although only a small positive charge was reached for DTEABr. These results point out that, with the exception of 11PyBr, surfactant/ctDNA complexes were formed.

**Figure 2:**
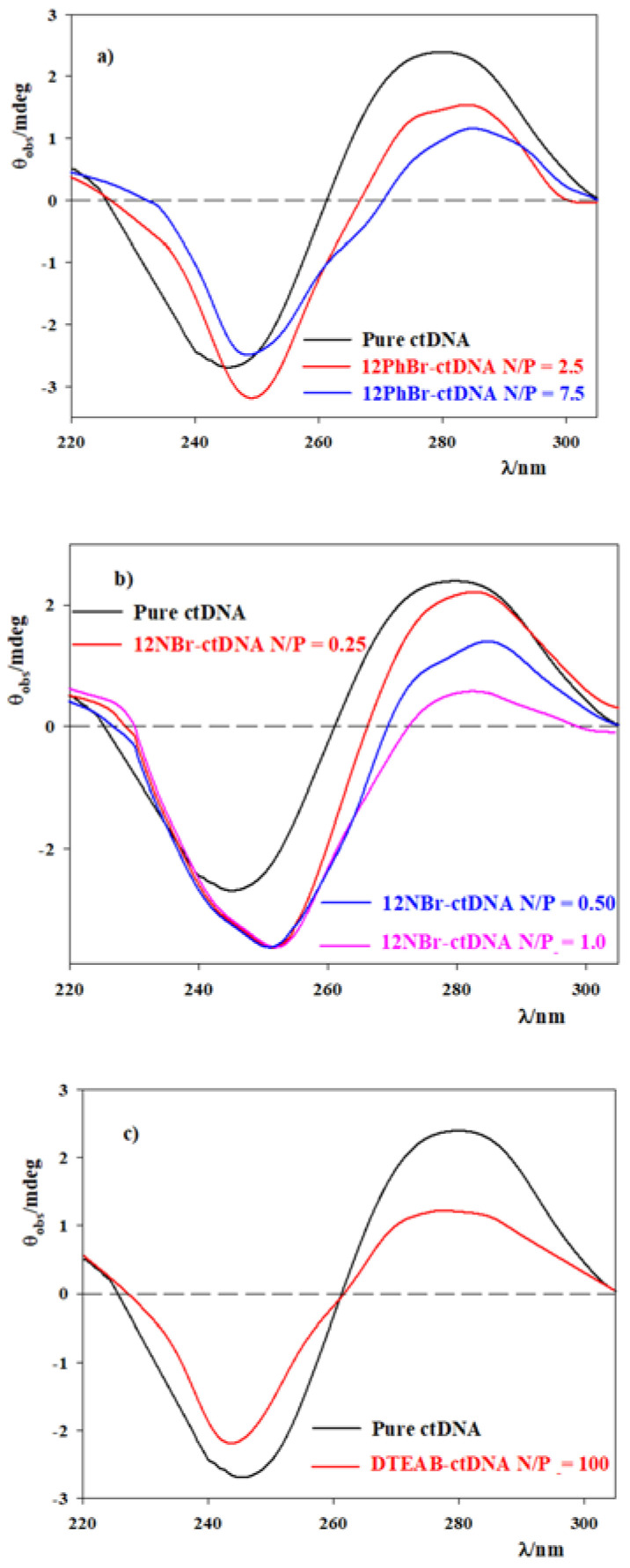
Dependence of the circular dichroism spectra of ctDNA (3.0 × 10^−5^ M) on the charge ratio N/P. The experimental values are expressed as the mean ± SD (*n* = 9). Experimental conditions: [ctDNA] = 3.0 × 10^−5^ M, HEPES 10 mM (pH = 7.4) and T = 303.0 ± 0.1 K. (**a**) 12PhBr/ctDNA, (**b**) 12NBr/ctDNA and (**c**) DTEAB/ctDNA.

One way of studying the DNA conformational changes is by circular dichroism (CD) [57]. Figure 2 shows the CD spectra registered in buffered surfactant/ctDNA solutions (HEPES 10 mM). It was ensured that the presence of the buffer or the surfactants in the solutions did not contribute to the spectra. The CD spectrum of pure DNA in Figure 2 corresponded to that for the right-handed B form of a double stranded nucleic acid at pH 7.4: a negative band close to 247 nm due to the right handed helicity of the polynucleotide, and a positive band at around 280 nm due to the stacking interactions between the base pairs [57]. For 12PhBr and 12NBr a displacement of the bands to longer wavelengths was observed, together with and a strong decrease in the intensity of the positive band. For 12NBr an increment in the intensity of the negative band was observed for all the N/P values studied, whereas for 12PhBr the negative band intensity remained approximately constant within the N/P range investigated. The formation of highly condensed DNA phases is usually related with these experimental observations [40]. In the case of the DTEABr solutions, small changes in the spectrum of pure ctDNA were observed, even for high N/P values, indicating that no important conformational changes in the nucleic acid occurred. No changes in the spectrum of pure DNA were observed in the presence of 11PyBr, even at high N/P charge ratio. Therefore, this surfactant did not cause conformational changes in the DNA. Therefore, the CD results seem to indicate that DTEABr and 11PyBr did not compact the polynucleotide, while 12PhBr and 12NBr varied its conformation due to their strong interactions with ctDNA.

**Figure 3:**
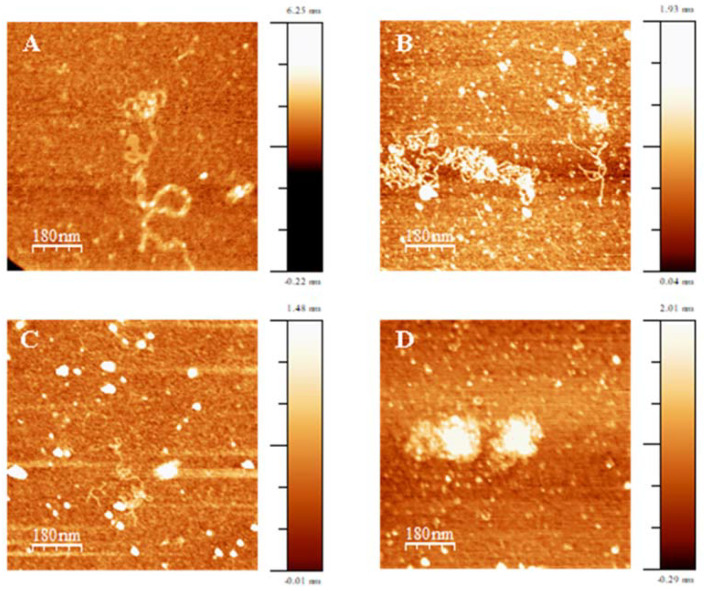
AFM topographic images of 12NBr/ctDNA buffered solutions, in 10 mM HEPES (pH = 7.4), adsorbed on APTES modified mica surface. [ctDNA] = 0.6 μM. (**A**) Pure ctDNA; (**B**) N/P =0.5; (**C**) N/P = 0.9; (**D**) N/P = 3.0.

This hypothesis can be confirmed by visualizing the surfactant/ctDNA complexes through AFM measurements. Figure 3 shows the AFM images obtained for 12NBr/ctDNA buffered solutions, and Figure S1 (Supplementary Material) presents the results corresponding to 12PhBr/ctDNA buffered solutions. One can see in Figure 3A the elongated form of pure ctDNA. When the amount of surfactant increased, some globular structures appeared and only globular forms were seen at high charge ratio N/P. Similar images were found for the 12PhBr/ctDNA solutions (Figure S1, Supplementary Material). These results confirm conformational changes of the nucleic acid, and a total condensation in the surfactant/ctDNA complexes formed. Similar results were previously observed for several cationic surfactants/ctDNA systems [11,12,13,14,20,21,22,25,33,34,37,40,42].

Two main processes can define the surfactant-DNA interactions: (i) surfactant cationic head groups electrostatic interactions with the DNA phosphates resulting in the displacement of the DNA sodium counter-ions from the nucleic acid surface into the solution; (ii) as surfactant binding progresses, hydrophobic interactions between the incoming surfactant molecules and the layer of surfactant molecules already bound to the nucleic acid become more important, which brings multiple DNA molecules together [11]. This explains the high degree of cooperativity observed in the binding isotherms. All the surfactants considered had a 1+ charge; therefore the electrostatic attractive interactions were expected to be similar for all of them. However, they showed different tendencies to self-aggregate, as shown by their cmc values, summarized in [11] and in Table S1 (Supplementary Material). A simple approach to estimate the apparent equilibrium binding constant of a surfactant to DNA, K_app_, is using Equation (3):

(3) $$K_{\mathrm{EB}}\left[\mathrm{EB}\right]=K_{\mathrm{pp}}\left[{\mathrm{Surfactant}}_{50\%}\right]$$

**Figure 4:**
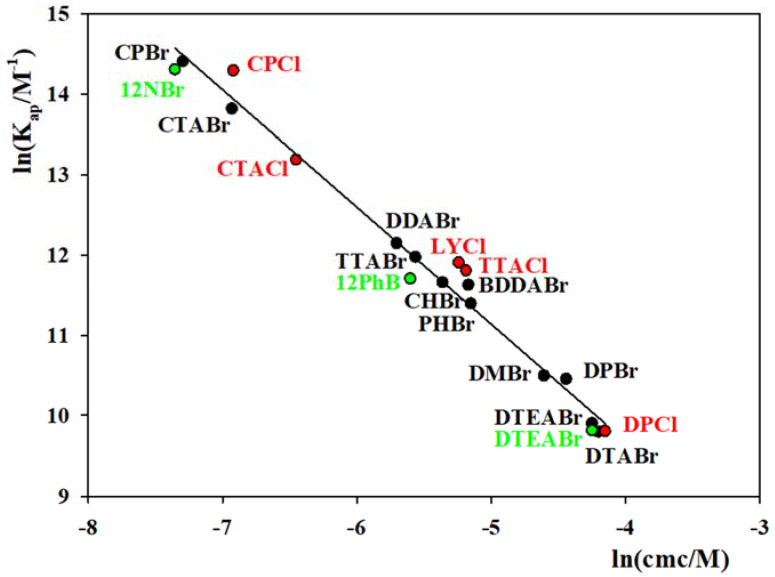
Plot of ln(Kapp) vs. ln(cmc) for the different surfactants investigated. Black circles: bromide surfactants; red circles: chloride surfactants; green circles: bromide surfactants investigated in this work. T = 303 K. CTABr: Hexadecyltrimethylammonium bromide; TTABr: Tetradecyltrimethylammonium bromide; DTABr: Dodecyltrimethylammonium bromide; CTACl: Hexadecyltrimethylammonium chloride; TTACl: Tetradecyltrimethylammonium chloride; CPBr: Hexadecylpyridinium bromide; DDPBr: Dodecylpyridinium bromide; CPC: Hexadecylpyridinium chloride; DPC: Dodecylpyridinium chloride; BDDBr: Benzyldimethylammonium bromide; DMBr: 1-Dodecyl-3-methylimidazolinium bromide; DTEABr: Dodecyltriethylammonium bromide; LYCl; (*S*)-5-Acetamido-6-(dodecylamino)-*N*,*N*,*N*-trimethyl-6-oxohexan-1-ammonium chloride; PHBr: N-Benzyl-*N*,*N*-dimethyl-*N*-(1-dodecyl)ammonium bromide; CHBr: N-Cyclohexyl-*N*,*N*-dimethyl-n-(1-dodecyl)ammonium bromide; DDABr: [2-(2-benzoylammino-etoxy)ethyl]-*N*-dodecyl-*N*,*N*-dimethyl-ammonio bromide; 12PHBr; Triethyl(1-phenoxydodecyl)ammonium bromide; 12NBr: Triethyl(2-naphthoxydodecyl)ammonium bromide.

In this equation [EB] is the ethidium bromide concentration present in the competitive binding experiments, K_EB_ is the equilibrium binding constant of the dye to the nucleic acid [58] and [surfactant_50%_] is the surfactant concentration corresponding to a 50% reduction in the fluorescence intensity of DNA-bound EB. Figure 4 shows the plot of ln(K_app_) versus ln(cmc) for the surfactants investigated in [11] (black and red circles) and those studied in this work (green circles). One can see that a good linear correlation was observed. This result indicates that the cmc can be used as a criterion to predict the DNA-compacting capacity of the surfactants considered. If one takes into account that the driving force of the micellization process is the hydrophobic contribution [59], the observed linear correlation makes evident that the binding strength of the cationic surfactants investigated in this work, as well as those studied in [11], to ctDNA is mainly controlled by their self-association tendency. Figure 4 shows that counter-ion nature influences surfactant DNA-compacting capacity. The bromides are better condensing agents that chlorides due to a higher hydrophilicity of the chloride ion as compared to the bromide ion, which results in a lower reduction of the electrostatic repulsions between the cationic surfactant head groups at the micellar interface. As a consequence, the cmc of chloride surfactants is higher than those of bromide surfactants. Figure 4 also shows that single-chained surfactants with functional groups at the end of the hydrophobic tail, and those without these groups, seem to behave similarly, although more surfactants with functional groups at the end of the hydrophobic tail should be investigated in order to confirm this assertion.

Lower there is one issue related to Figure 4 that needs explanation. This figure shows two ln(K_app_) values for DTEABr, even if they are really close. The black circle value corresponds to a HEPES 40 mM buffered solution at pH = 7.4, whereas the green circle value corresponds to a HEPES 10 mM buffered solution at pH = 7.4. The fact that the K_app_ value was practically the same, within experimental errors, indicates that the change in the HEPES concentration did not affect the surfactant/DNA interactions, at least within the concentration range studied.

### 3.2. DOPE/12NBr/pDNA Lipoplexes

Gene therapy is the focus of many investigations carried out nowadays because it has been shown to be a promising option to treat several acquired diseases such as AIDS, cancer or genetic disorders [60,61,62,63,64,65,66,67]. Due to the biological barriers of the human body against foreign substances, it has been necessary to prepare biodegradable and biocompatible nanovehicles, called vectors, to protect the genetic material. Vectors can be viral and nonviral. The more frequently used nonviral vectors are micelles, cyclodextrins, nanoparticles, calixarenes and liposomes [68,69,70,71]. Cationic liposomes are good nonviral vectors because they avoid degradation of the genetic material by nucleases, and their positive charge overcomes the electrostatic repulsions between the DNA phosphate groups and the negative charges of the cell lipid bilayer, making genetic material delivery more favorable [69,72,73,74,75]. With this in mind, DOPE/12NBr liposomes were prepared and characterized. 12NBr was chosen because it was the most efficient DNA-compacting agent investigated in this work (see Figure 1 and Figure 3). Since 12NBr can completely condense the nucleic acid at low surfactant concentration, it would be expected that the liposomes prepared would show higher cell viability. The lipoplexes were prepared with the plasmid pEGFP-C1, pDNA. The goal of these studies was to investigate the transfection efficiency (TE) of these nanovehicles.

**Figure 5:**
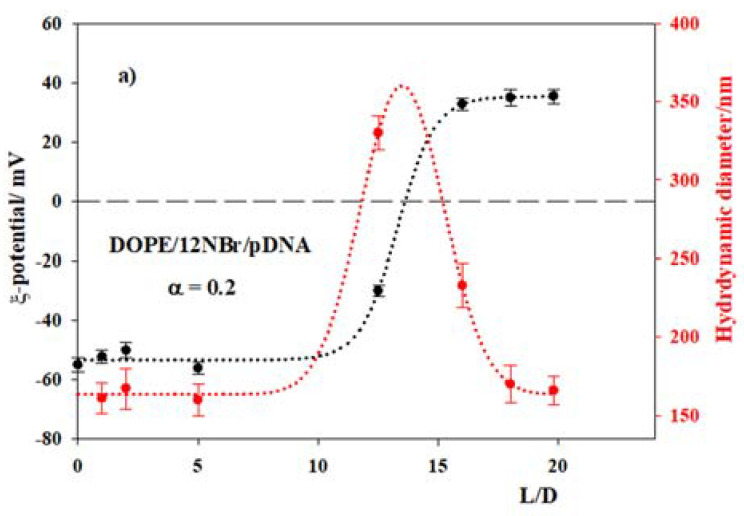

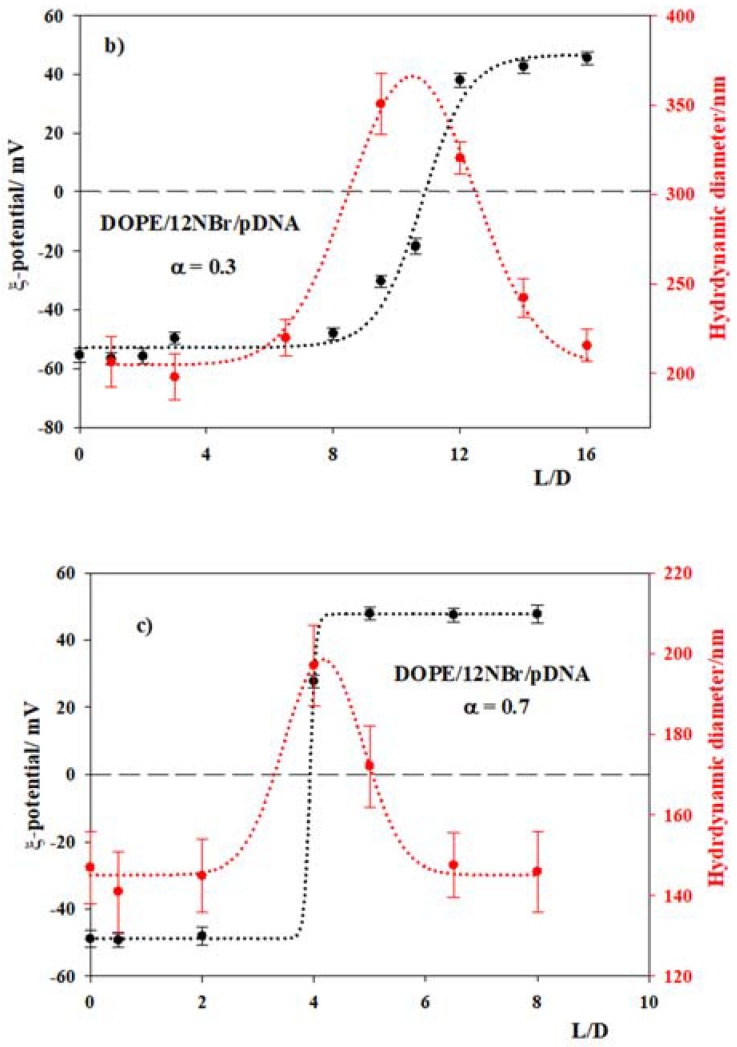
Dependence of the zeta potential and of the hydrodynamic diameter of DOPE/12NBr/pDNA liposomes on the mass ratio, L/D, for various cationic surfactant molar fractions, α, in 10 mM HEPES (pH = 7.4) at T = 303.0 ± 0.1 K. (**a**) α = 0.2, (**b**) α = 0.3 and (**c**) α = 0.7.

The DOPE/12NBr/pDNA lipoplexes, with different cationic surfactant molar fractions, α, and mass ratio, L/D, were prepared as described in the Experimental Section. Figure 5 shows the dependence of the zeta potential and of the hydrodynamic diameter of the lipoplexes on the mass ratio, L/D, for various cationic surfactant molar fractions, α, in 10 mM HEPES (pH = 7.4). In all cases a charge inversion was observed upon increasing L/D; that is, when the amount of cationic surfactant 12NBr increased. The L/D value for which the zeta potential of the lipoplex was zero, approximately corresponding, within experimental errors, to the maximum hydrodynamic diameter. This is an expected result since no electrostatic repulsions take place for neutral, or close to neutral, lipoplexes and, as a result, aggregation of these nanostructures occurs. The effective charge, Z_eff_ of the lipoplexes is defined by Equation (4):

(4) $$Z_{\mathrm{eff}}=\frac{n_{+}}{n_{-}}$$

n_+_ and n_−_ being the moles of positive and negative charges, respectively. The L/D value corresponding to an effective charge equal to 1 is that for which the lipoplexes are neutral. Figure 5 shows that Z_eff_ was equal to 1 at lower L/D values when the cationic surfactant molar fraction α was higher. This can be explained considering that, in all the experiments, the amount of pDNA remained constant and an increase in α meant an increment in the amount of the cationic surfactant. Therefore, a lower L/D value would be necessary to neutralize the negative charges of the nucleic acid.

**Figure 6:**
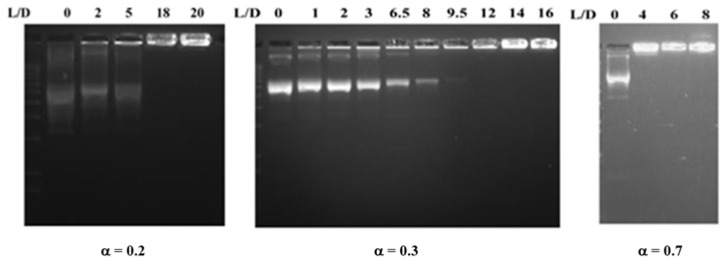
Electrophoretic mobility shift assay on an agarose gel (1%) for DOPE/12NBr/pDNA lipoplexes at different α values. Experiments were run in TAE buffer (40 mM tris-acetate, 1 mM EDTA) and stained with Red Safe. Electrophoresis was performed at 90 V until completion.

Figure 6 shows the results obtained by agarose gel electrophoresis. The experimental observations in this figure are in agreement with those shown in Figure 5.

One can see that an increase in the cationic surfactant molar fraction α resulted in the migration of pDNA being hindered at lower L/D values. That is, the nucleic acid was neutral or positively charged at at lower L/D values the higher α is.

**Figure 7:**
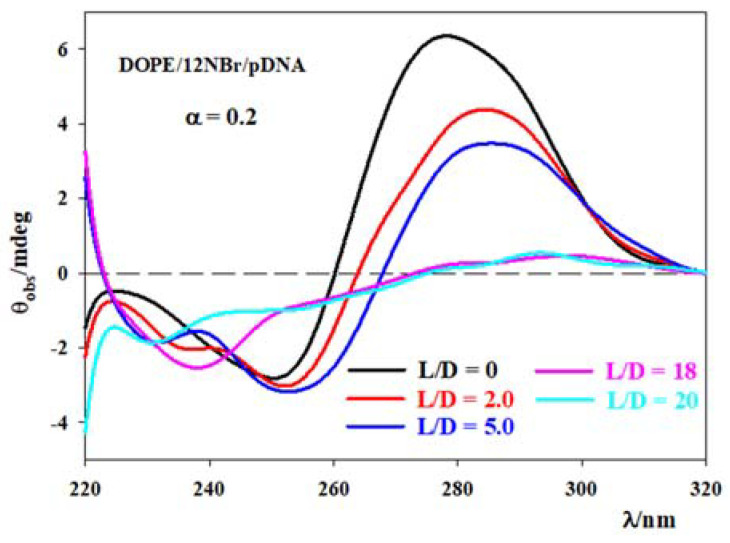

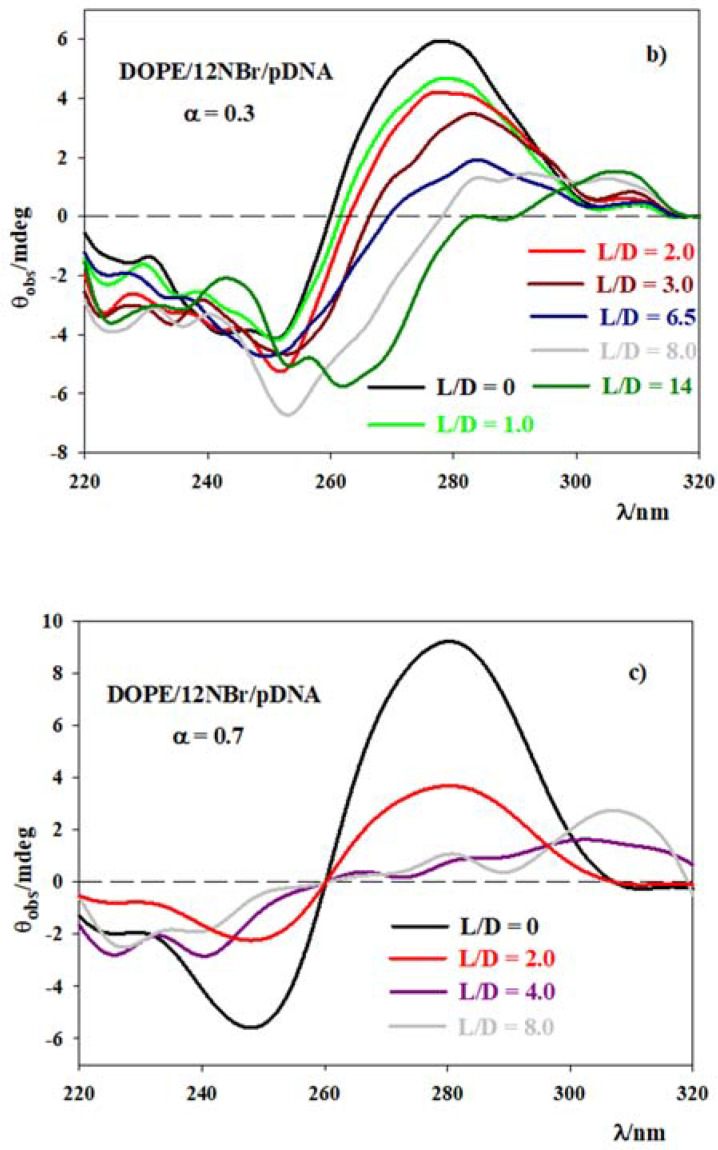
Dependence of the circular dichroism spectra of pDNA (3.0 × 10^−5^ M) in the DOPE/12NBr/pDNA lipoplexes, for various α values, on the ratio L/D. The experimental values are expressed as the mean ± SD (*n* = 9). Experimental conditions: [pDNA] = 3.0 × 10^−5^ M, HEPES 10 mM (pH = 7.4) and T = 303.0 ± 0.1 K. (**a**) α = 0.2, (**b**) α = 0.3 and (**c**) α = 0.7

In order to investigate if the formation of the DOPE/12NBr/pDNA lipoplexes was accompanied by a morphological change of the pDNA, CD spectra were registered for various α at different L/D values. Figure 7 shows that the changes in the pDNA CD spectra depend on α. For L/D = 0, that is, when no cationic surfactant was present, the typical CD spectrum of a right-handed B form of a double stranded nucleic acid was observed [59]. For α = 0.2, a displacement of the bands to longer wavelengths, a small increase in the intensity of the negative band and a strong decrease in the intensity of the positive band were observed for L/D < 5. At high L/D values, 18 and 20, both bands practically disappeared. In the case of α = 0.3 a shift of the bands to longer wavelengths was observed, together with a strong decrease of the positive and negative bands. Finally, for α = 0.7, for all the L/D values investigated, similar observations to those for α = 0.2 at high L/D values were found, although the intensity of the negative band remained approximately constant. The variations in the CD spectra found for all the lipoplexes solutions studied was in accordance with a compaction of pDNA and a partial denaturation of the double strand [11,25,31,34,40,42,75]. It is worth noting that solubility problems precluded experiments at higher L/D ratios for the α values investigated. For all the cationic surfactant molar fractions α studied, an increase in L/D, that is, an increase in the amount of 12NBr within the lipoplexes, resulted in compaction of the plasmid DNA.

**Figure 8:**
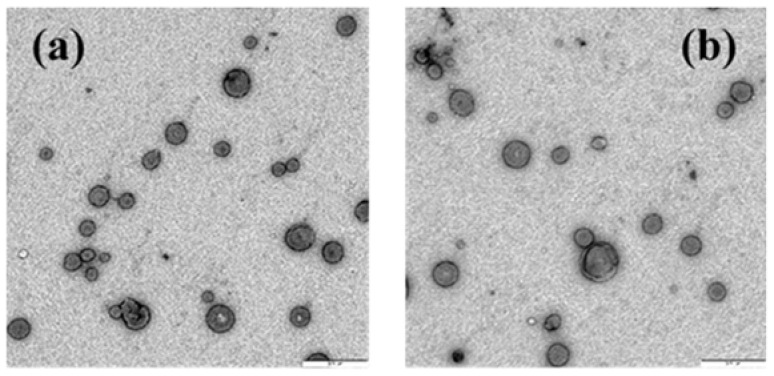
TEM images, obtained by negative staining, of: (**a**) liposomes at α = 0.7, and (**b**) lipoplexes at α = 0.7 and L/D = 8 (Scale bar = 0.5 µm).

The morphology of the liposomes and lipoplexes was investigated using TEM. Two examples of TEM images are shown in Figure 8. The corresponding size distribution histograms are shown in Figure S2 (Supplementary Material). The average size of the lipoplexes with α = 0.7 and L/D = 8 shown in Figure 8b, measured from several TEM images corresponding to different samples, was 160 ± 52 nm, which is agreement, within experimental errors, with that estimated using DLS measurements, shown in Figure 8. For lipoplexes with α = 0.2 and L/D = 20 and α = 0.3 and L/D = 16 the hydrodynamic diameter found by TEM were 178 ± 46 nm and 203 ± 55 nm, respectively, in agreement with those estimated by DLS, within experimental errors. The morphology of both the liposomes and the lipoplexes, was found to be spherical for all the samples investigated. In all cases, the chosen L/D values corresponded to cationic lipoplexes, where the nucleic acid was compacted, which are two characteristics required for a potential efficient vector [4,5].

### 3.3. Transfection Efficiency of DOPE/12NBr/pDNA Lipoplexes

**Figure 9:**
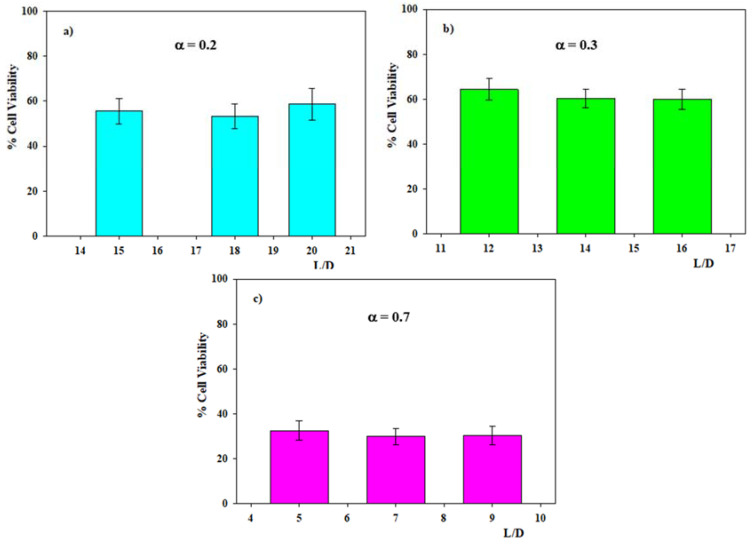
Dependence of the cell viability (%) of the DOPE/12NBr/pDNA lipoplexes on L/D in U2OS cancer cell line, at 48 h, for different cationic surfactant molar fractions α, (**a**) α = 0.2, (**b**) α = 0.3 and (**c**) α = 0.7.

First, information about the influence of 12NBr concentration on cell viability was obtained using the MTT assay r the human bone osteosarcoma epithelial cells U2OS, which is the cell line used in the transfection experiments. Figure 9 shows the results obtained. The L/D values chosen corresponded to the region where the charge of the lipoplexes was positive, in order to make their crossing through the negatively charged cellular membranes easier. The results show that the cell viability was much higher for α = 0.2 and α = 0.3 than for α = 0.7. On this basis, the transfection experiments were done using lipoplex solutions with α equal to 0.2 and 0.3 at various L/D ratios.

**Figure 10:**
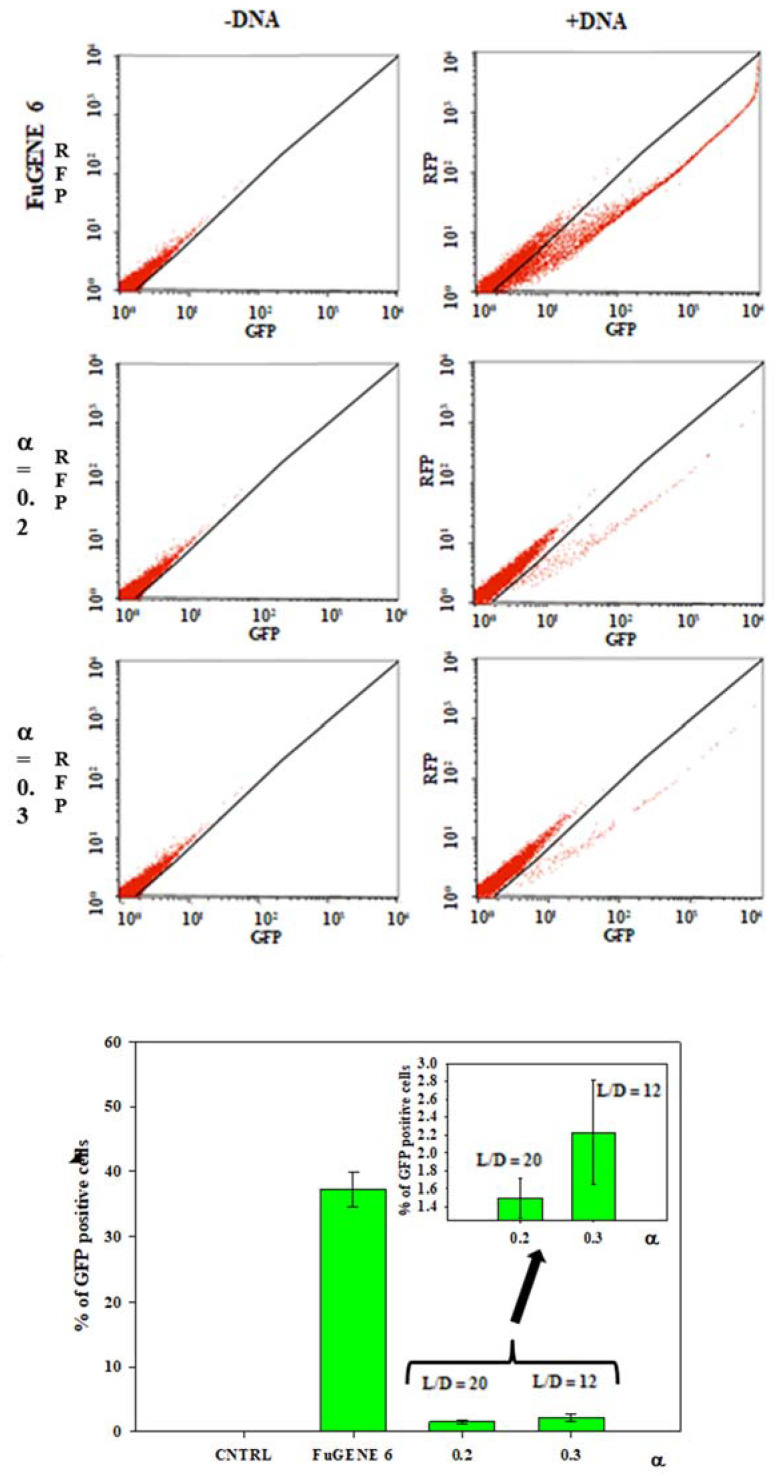
GFP expression of pEGFP-C1 in U2OS cells, using DOPE/NBr/pDNA lipoplexes at α = 0.2 and L/D = 20 and at α = 0.3 and L/D = 12, sorted on the basis of fluorescence at 24 h, post-transfection. CNTRL represents the negative control. FuGENE 6 was used as a positive control. The inset plot shows an enlarged version of the results obtained for α = 0.2 and L/D = 20 and α = 0.3 and L/D = 12.

**Figure 11:**
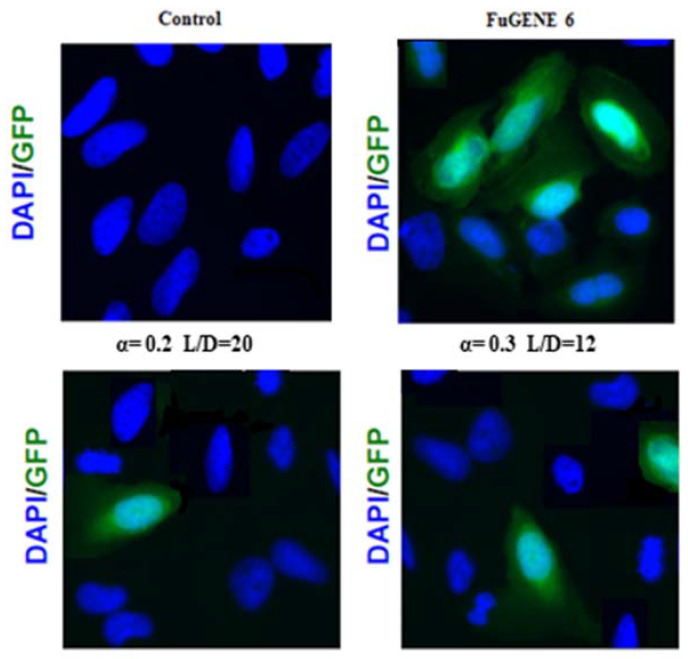
Representative images of GFP positive cells after transfection with 3 μg of pDNA with DOPE/NBr/pDNA lipoplexes at α = 0.2 and L/D = 20 and at α = 0.3 and L/D = 12.

The transfection process of the plasmid pEGFP-C1 was carried out on the U2OS cells because this is an easy-to-transfect cell line. Figure 10 presents the percentage of GFP cells with 3 μg pEGFP-C1 in the U2OS cell line. In this Figure, the TE of the transfection reagent FuGENE 6 is also shown for the sake of comparison. The same result can be observed in Figure 11, where representative images of GFP-positive cells after transfection are shown. Expression of GPF is a common readout of transfection which does not require any additional manipulation of the sample, because the GFP is an intrinsically fluorescent protein, so the fluorescence can be readily observed directly. The transfection was carried out for a given α value at the three L/D ratios shown in Figure 9. The TE was negligible for α = 0.2 and L/D equal to 15 and 18. Similar results were found for α = 0.3 and L/D equal to 14 and 16. The amount of pDNA was changed in order to investigate if the concentration of plasmid present in the lipoplexes solutions could influence on the TE observed. No substantial changes were found. A transfection efficiency equal to 1.5% and 2.5% was obtained for the DOPE/12NBr/pDNA lipoplexes with α = 0.2 and L/D = 20 and α = 0.3 and L/D = 12, respectively. The small increase in the TE of the liposomes with α = 0.3, compared to the TE of those with α = 0.2, could be due to the higher cell viability shown by the former with respect to the latter (see Figure 9). The transfection efficiency of the lipoplexes with α = 0.2 and L/D > 20, and α = 0.3 and L/D > 12, could not be carried out because of solubility problems, as mentioned above. In spite of the low TE obtained for the DOPE/12NBr/pDNA lipoplexes investigated, the observation of a positive transfection efficiency compared to the negative control, CNTRL, (see Figure 10), confirmed the possibility of using similar lipoplexes as vectors in gene therapy. Further studies to improve the transfection process through changes in the structure of the cationic surfactant are in progress. In this regard, the variation of the hydrophobic chain length is interesting. Previous results have shown that for a given cationic surfactant there is an optimum tail length for which the TE is maximum [76,77,78]. This could be related to the formation of a more compact lipid bilayer in the lipoplexes, making the delivery of the genetic material easier. This would result in a less favorable breaking of the lipoplexes as well as in a more difficult escape of the plasmid to the cytosol, thereby making the transfection process more favorable.

## 4. Conclusions

In this work the DNA-compacting efficiency of single-chained surfactants with functional groups at the end of the hydrophobic tail was investigated. The results show that, as in the case of single-chained surfactants without these groups, the surfactant tendency of self-aggregation is the main factor determining their capacity to condense the nucleic acid. This is made evident by the linear correlation between ln(K_app_) vs. ln(cmc), where K_app_ is the apparent binding equilibrium constant of the surfactants to the DNA. That is, the hydrophobic interactions play a key role in the surfactant/nucleic acid interactions.

DOPE/surfactant/pEGFP-C1 lipoplexes of different compositions were prepared using 12NBr, the surfactant with the highest compacting capacity among those studied. The presence of a low surfactant concentration in the lipoplexes was an interesting aspect because it could result in an increase of the cell viability of these nanovehicles in several cell lines. The lipoplexes were prepared by varying the cationic surfactant molar fraction α and changing the mass ratio L/D for each α value. Using several techniques, they were characterized, the results showing the charge inversion of the plasmid pEGFP-C1 and its subsequent compaction when L/D increased, for a given α value. The charge inversion was observed at lower L/D ratios the higher the α value is. This compaction was a prerequisite for gene transfection. Cytotoxicity studies showed that for α values equal to 0.2 and 0.3, changing the L/D ratio, the cell viability for human bone osteosarcoma epithelial cells U2OS was close to 60%.

The transfection process of the plasmid pEGFP-C1 was carried out on U2OS cells using lipoplexes at different cationic surfactant molar fractions α, varying the L/D mass ratio. Only for DOPE/12NBr/ pEGFP-C1 lipoplexes with α = 0.2 and L/D = 20 and α = 0.3 and L/D = 12 was transfection observed, the TE being equal to 1.5% and 2.5%, respectively. These TE values are low, but they point out that similar lipoplexes could be more efficient vectors. Changes in the structure of the surfactant will be necessary to improve the TE. One of the strategies to follow is to vary the length of the hydrophobic chain because, for a given cationic surfactant, there is an optimum tail length for which the TE is maximal [76,77,78].

## Supplementary Materials

The following are available online at https://www.mdpi.com/article/10.3390/pharmaceutics13040589/s1, Figure S1: AFM topographic images of 12PhBr/ctDNA buffered solutions, in 10 mM HEPES (pH = 7.4), adsorbed on APTES modified mica surface. [ctDNA] = 0.6 μM. (A) N/P = 9; (B) N/P = 25. Figure S2: Histograms generated using the size distribution tool of the ImageJ bundled with 64-bit Java 1.8.0_172 software of the TEM microscope for: (A) liposomes at α = 0.7, and (B) lipoplexes at α = 0.7 and L/D = 8. Table S1: Values of the critical micellar concentration of the surfactants investigated in this work.
