# Supplementary material for: Cationic Single-Chained Surfactants with a Functional Group at the End of the Hydrophobic Tail DNA Compacting Efficiency
Source: Pharmaceutics. 2021 Apr 20;13(4):589. doi: 10.3390/pharmaceutics13040589 (PMC8074900; doi:10.3390/pharmaceutics13040589)
Supplement: Supplementary file 1 [file pharmaceutics-13-00589-s001.zip › pharmaceutics-1140078-supplementary.pdf]

pharmaceutics

Supplementary Materials: Cationic Single-Chained Surfactants with a Functional Group at the End of the Hydrophobic Tail DNA Compacting Efficiency

José Antonio Lebrón Pilar López-Cornejo Elena García-Dionisio Pablo Huertas Margarita García-Calderón María Luisa Moyáa Francisco José Ostos and Manuel López-López

Table S1. Values of the critical micellar concentration of the surfactants investigated in this work, taken from refs. 47-49. The cmcs were determined by conductivity.

Surfactant	DTEABr	12PhBr	12NBr	11PyBr[a]
cmc/mM	14.3 ± 0.4	3.7 ± 0.2	0.641 ± 0.015	---

[a]This surfactant does not self-aggregate.

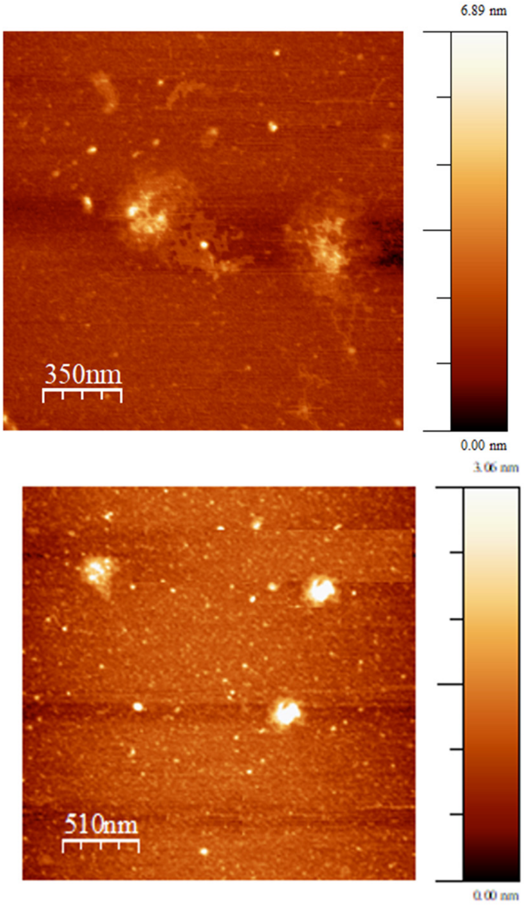

Figure S1. AFM topographic images of 12PhBr/ctDNA buffered solutions, in 10 mM HEPES (pH = 7.4), adsorbed on APTES modified mica surface. [ctDNA]= 0.6 μM. **A**) N/P = 9; **B**) N/P =

Citation: Lebrón, J.A.; López-Cornejo, P.; García-Dionisio, E.; Huertas, P.; García-Calderón, M.; Moyá, M.L.; Ostos, F.J.; Cationic Single-Chained Surfactants with a Functional Group at the End of the Hydrophobic Tail DNA Compacting Efficiency. *Pharmaceutics* **2021**, *13*, 589. https://doi.org/10.3390/pharmaceutics13040589

Academic Editors: Gert Fricker and Ian S. Blagbrough

Received: 23 February 2021
Accepted: 15 April 2021
Published: 20 April 2021

Publisher's Note: MDPI stays neutral with regard to jurisdictional claims in published maps and institutional affiliations.

Copyright: © 2021 by the authors. Licensee MDPI, Basel, Switzerland. This article is an open access article distributed under the terms and conditions of the Creative Commons Attribution (CC BY) license (http://creativecommons.org/licenses/by/4.0/).

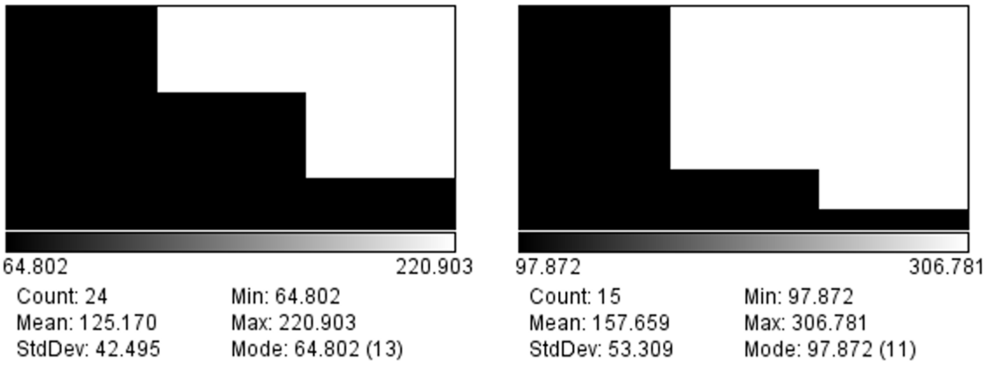

(A) **(B)**

25.

Figure S2. Histograms generated using the size distribution tool of the ImageJ bundled with 64-bit Java 1.8.0_172 software of the TEM microscope for: (**A**) liposomes at α = 0.7, and (**B**) lipoplexes at α = 0.7 and L/D = 8.
